# BRAF Mutation Analysis: A Retrospective Evaluation of 8365 Diagnostic Samples with a Special View on Canine Breeds (2018–2024)

**DOI:** 10.3390/vetsci12080729

**Published:** 2025-08-02

**Authors:** Marielle Appenzeller, Alexandra Kehl, Katrin Törner, Katharina Charlotte Jensen, Robert Klopfleisch, Heike Aupperle-Lellbach

**Affiliations:** 1 LABOKLIN GmbH & Co. KG, 97688 Bad Kissingen, Germany; kehl@laboklin.com (A.K.); toerner@laboklin.com (K.T.); 2 School of Medicine, Institute of Pathology, Technical University of Munich, 80333 München, Germany; 3 Institute for Veterinary Epidemiology and Biostatistics, School of Veterinary Medicine, Free University of Berlin, 14163 Berlin, Germany; charlotte.jensen@fu-berlin.de; 4 Institute of Veterinary Pathology, Free University of Berlin, 14195 Berlin, Germany; robert.klopfleisch@fu-berlin.de

**Keywords:** dog, *BRAF* V595E mutation, urothelial carcinoma, transitional cell carcinoma, signalment

## Abstract

The *BRAF* V595E mutation analysis in canine urothelial carcinomas (UCs) has found its way into routine diagnostics, but no data analysis has been published until now. The present study aimed to estimate the distribution of age, sex, and breed in 8365 canine diagnostic samples submitted for *BRAF* mutation analysis during 2018–2024. The specimens included 8215 urine samples, 17 cytological, and 133 histopathological specimens, and were submitted in cases of suspected UC, to rule out UC, or for screening purposes. All samples were tested for the *BRAF* V595E mutation using droplet digital PCR (ddPCR). The data were statistically analysed and logistic regression models (Odds Ratio (OR)) were calculated. Compared to samples from mixed-breed dogs, the specimens from Scottish Terriers (OR: 4.21), Shetland Sheepdogs (OR: 2.65), Beagles (OR: 2.33), Fox Terriers (OR: 1.92), Staffordshire Bull Terriers (OR: 1.86), Magyar Vizslas (OR: 1.77), Chihuahuas (OR: 1.70), and West Highland White Terriers (OR: 1.43) had a significantly increased probability of the presence of *BRAF* mutation indicating UC. The youngest *BRAF*-positive dogs of these predisposed breeds (*n* = 4) were 5 years old. In conclusion, screening tests in predisposed breeds may be recommended from the age of 5 years.

## Simple Summary

In approximately 80% of cases of canine urothelial carcinoma (UC), the mutation V595E in the *BRAF* gene is present. As a positive *BRAF* mutation analysis is conclusive for canine UC, it is used as a diagnostic tool to identify UC patients and is the basis for targeted therapy. The present study aimed to analyse our data over six years (2018–2024) from canine samples submitted for *BRAF* mutation analysis, mostly from across Europe, to assess the distribution of age, sex, and breed. Sample material from the urinary tract of 8365 dogs was examined for the *BRAF* mutation using droplet digital PCR (ddPCR). These retrospective data were statistically analysed, and logistic regression models were calculated. Compared to samples from mixed-breed dogs, the specimens from Scottish Terriers, Shetland Sheepdogs, Beagles, Fox Terriers, Staffordshire Bull Terriers, Magyar Vizslas, Chihuahuas, and West Highland White Terriers had a significantly increased probability of the presence of the *BRAF* mutation, indicating UC. The youngest *BRAF*-positive dogs of these predisposed breeds (*n* = 4) were 5 years old. In conclusion, screening tests in predisposed breeds may be recommended from the age of 5 years.

## 1. Introduction

The most common reason for death in dogs is cancer [1,2]. Neoplasms of the lower urinary tract represent <2% of all malignant tumours in dogs [3,4]. Urothelial carcinoma (UC), also referred to as transitional cell carcinoma (TCC), is the most common malignant tumour of the urinary tract in dogs [4]. UC is an aggressive tumour with metastatic potential, as more than 90% of UC cases are intermediate to high-grade and invasive [4,5,6]. Metastases to regional lymph nodes and distant metastases in the lungs and abdominal organs were detectable in 14% and 16% of dogs, respectively, at the time of diagnosis, and in 49% at the time of death [7,8]. Distant metastases occur frequently in the lungs and abdominal organs [9] and bone [10].

A breed-specific risk for canine UC is known, especially for Scottish Terriers, as they have an increased risk (19 times higher) of developing UC compared to mixed breeds [7]. A breed predisposition is also present in Shetland Sheepdogs, Beagles, Fox Terriers, and West Highland White Terriers [4,7]. In addition, the summary of analyses of the Veterinary Medical Data Base, a database of case information from multiple university veterinary teaching hospitals in the United States and Canada, compared cases of dogs with and without UC between 1999 and 2010 [9]. These analyses confirmed the risk for UC in previously mentioned breeds and also indicated risk in additional breeds like American Eskimo Dog, Keeshond, Samoyed, and Dalmatian [9].

UC often correlates with nonspecific clinical symptoms of dysuria, such as strangury, haematuria, pollakiuria, and tenesmus [3,4,7,11,12]. These findings can also be associated with urolithiasis or lower urinary tract infections like cystitis. Thus, diagnostic tools are required to distinguish a neoplastic process from inflammation. A cytological or histological examination can indicate UC if atypical transitional cells with criteria of malignancy are present. However, inflammation and reactive changes can also induce cellular pleomorphism, which can complicate the differentiation [13,14].

Recently, tumour research and its various diagnostic possibilities, including molecular testing, are of increasing relevance [15]. Molecular biomarkers can assist not only in clinical diagnosis, but also in prognosis and therapeutic strategies [16].

The *BRAF* gene is one of the three isomers of the rapidly accelerated fibrosarcoma (RAF) protein family with serine/threonine kinase activity, which also comprises the isoforms *ARAF* and *CRAF* [17,18,19]. The somatic mutation leads to hyperactivation of the mitogen-activated protein kinase/extracellular regulated kinase (MAPK/ERK) pathway in humans and canines [20,21,22,23] and therefore to abnormal differentiation and cell proliferation [24,25]. In 66% of human malignant melanomas and a lower percentage in colon cancer and papillary thyroid cancer, the mutated *BRAF* gene (V600E) was identified [26]. Glu for Val substitution accounts for 90% of *BRAF* mutations in human cancers [27].

The canine V595E mutation of *BRAF* is homologous to the oncogenic human V600E mutation [28,29]. The V595E mutation of *BRAF* was detected in 67% [29], 75% [30], and in 87% [28] of canine UCs. The high *BRAF* mutation rate in canine UC makes *BRAF* mutation analysis a valid diagnostic option, but only *BRAF*-positive results confirm UC. A negative result of the mutation analysis can indicate that (1) the patient does not have a tumour, (2) the sample does not contain mutated cells, or (3) a UC without a V595E mutation on the *BRAF* gene is present. For the V595E mutation of *BRAF*, significant breed predisposition was found: 55% [31] to 86% [32] of the terriers with diagnosed UC tested positive for the *BRAF* mutation. No sex-specific predisposition to the *BRAF* mutation was identified in the terrier breeds [31,32].

The analysis of *BRAF* mutation has proven to be a valid diagnostic option or an addition to histopathological or cytological examination for canine UC diagnosis. The minimally invasive tool of identifying *BRAF* mutations from urine avoids an invasive sampling technique (e.g., biopsies) [13,30,33]. Additionally, biopsy sampling is often not possible due to the anatomically inaccessible location of the tumour tissue, the patient’s size and sex, the costs, and the clinician’s experience and equipment available in the clinic. Furthermore, cytology/histology may be limited by sampling quality [9,13,30].

The droplet digital PCR (ddPCR) assay for the detection of the canine *BRAF* V595E mutation, with a sensitivity of 83%, is helpful to diagnose UC in a non-invasive way or at an early stage of the disease [30]. No *BRAF* mutation was detected in non-neoplastic lower urinary tract cases, indicating a very high specificity (100%) of the test [28,30,33]. The positive *BRAF* result is considered conclusive, as none of the tumour-free dogs in the study cohort contained the *BRAF* mutation [33].

*BRAF* mutation analysis is used in routine diagnostics after the test was discovered for the early detection of UC by the Breen-Lab (Dept. of Molecular Biomedical Sciences, College of Veterinary Medicine) at North Carolina State University [30]. However, no major analyses of *BRAF* results from diagnostic dog samples have been published so far. This study aims (1) to evaluate the sample submissions from dogs mainly across Europe; (2) to analyse the signalment of dogs with a positive *BRAF* mutation signal.

## 2. Materials and Methods

The data sets from 8365 diagnostic samples submitted for *BRAF* mutation analysis in the years 2018 to 2024 to LABOKLIN GmbH & Co. KG, Germany were included in this study. The samples were submitted for diagnostic purposes, not for scientific research. Inclusion criteria were: (1) complete signalment (breed, age, and sex were indicated on the submission form) and (2) the result of *BRAF* mutation analysis was either positive or negative. The specific indication for each submission was not provided. Of the initial 9556 cases that underwent *BRAF* testing with complete signalment, non-sufficient or no DNA could be isolated in 1191 cases (12.5% of the cases). These cases were therefore excluded from further analysis.

Most of the samples (*n* = 4856, 58.1%) were derived from Germany, 41.5% of the specimens (*n* = 3472) were from other European countries, and 0.4% (*n* = 37) of the samples originated from China. Details of the Chinese samples can be found in the Supplementary Material (Table S1). Among the material submitted were 8215 urine samples, 17 cytological smears from fine needle aspirate biopsies or catheter aspiration biopsies, and 133 formalin-fixed, paraffin-embedded (FFPE) tissue samples. The FFPE tissue specimens correspond to urothelial biopsies from cases in which histopathological examination was inconclusive due to suboptimal sample quality. This was either due to squeeze artefacts or epithelial pleomorphism caused by severe inflammation.

The samples are identified as urothelial material based on the clinical report and localisation of sampling. The samples for the *BRAF* mutation test were submitted in cases of suspected UC, to rule out UC in the presence of clinical symptoms, or for screening purposes. The presence of urothelial prostate cells could not be completely excluded in smears of the urethra. It was not possible to differentiate between urothelial and prostate carcinoma by cytological or PCR examination of the submitted urine.

The breed names used in this study correspond to the Fédération Cynologique Internationale (FCI) breed nomenclature. If the FCI did not officially recognise the breed, it was classified as a mixed-breed dog together with those ‘not otherwise specified’ mongrels. The mixed breeds served as the reference for the statistical analyses.

As previously published, *BRAF* mutation analysis was performed on DNA isolated from each sample, targeting the c.1784 T > A mutation using ddPCR with a mutation-specific TaqMan^®^ assay, following the cBRAF V595E ddPCR protocol described by Mochizuki et al. [30]. The evaluation used the Droplet Reader (Bio-Rad, Feldkirchen, Germany) and the QuantaSoft™ software (Bio-Rad, Feldkirchen, Germany).

The data records were extracted from a customised database into an Excel file (Microsoft, www.microsoft.com), including signalment, sample material, and the results of *BRAF* mutation analysis. Descriptive analyses and multifactorial logistic regression models were calculated, including age (numeric), sex (M, male; MN, male neutered; F, female; FN, female neutered), and breed. Results of the multifactorial logistic regressions were presented in a forest plot and a heat map. After conducting a one-way ANOVA on the eight dog breeds with the highest proportions of positive *BRAF* results and the mixed-breeds, a Hochberg’s GT2 post hoc test was performed to carry out all pairwise comparisons between the groups. This test was chosen due to unequal group sizes and to control for Type I error across multiple comparisons. Pearson’s Chi-squared test was used to test associations between the categorical variables ‘sex’ and ‘result of *BRAF* mutation analysis’. Fisher’s exact test was used instead of the Chi-square test if the expected cell counts were too low to meet the assumptions of the Chi-square test. The significance levels are represented by stars (* *p* < 0.05; ** *p* < 0.01; *** *p* < 0.001). Statistical data analysis was performed using IBM SPSS Statistics, version 29.0.0.0 (241).

Four different data sets were used: (1) The complete data set (*n* = 8365), to analyse the impact of age and sex on the results of the *BRAF* mutation analysis. (2) A data set (*n* = 7162) including all breeds with 25 or more individuals to estimate the effect of the breed in more statistical detail. Age and sex were also included to adjust for this. (3) A data set (n = 1339) including the non-terrier breeds with the highest and lowest proportion of *BRAF*-positive results. (4) A data set (*n* = 1137) including only the terrier breeds with 25 dogs or more to analyse these breeds in more detail.

## 3. Results

### 3.1. Complete Data Set

The complete data set contained samples from 2469 intact and 1930 neutered males as well as from 1597 intact and 2369 neutered females (Table S1). The mean age was 10.39 ± 2.93 years. In addition to the mixed-breed dogs, samples from a total of 230 FCI-recognised dog breeds were submitted. Besides the mixed-breed dogs (27.8%, *n* = 2326), the most frequent breeds of the submitted samples included Labrador Retrievers (5.6%, *n* = 469), Jack Russell Terriers (3.8%, *n* = 316), Beagles (2.9%, *n* = 245), Yorkshire Terriers (2.7%, *n* = 224), and French Bulldogs (2.5%, *n* = 208).

Among all the 8365 submitted samples, the canine V595E mutation on the *BRAF* gene was found in 2381 samples (28.5%). The remaining 5984 samples were tested *BRAF*-negative. Of the 37 samples submitted from China, six dogs were diagnosed with the *BRAF* mutation (16.2%). The breed distribution is similar to that of the European dogs in the cohort of this study. Therefore, no further distinction between the countries of origin is made in the following.

**Figure 1:**
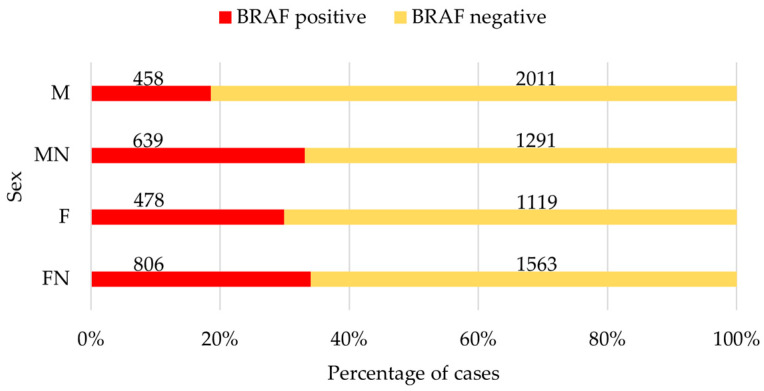
*BRAF* mutation was present in 18.6% of samples from intact male dogs and 33.1% of samples from neutered male dogs, as well as in 29.9% of samples from intact female dogs and 34.0% from neutered female dogs. M, male; MN, male neutered; F, female; FN, female neutered.

The percentage of *BRAF*-positive results was higher in neutered dogs of both sexes (MN = 33.1%; FN = 34.0%) than in their intact counterparts (M = 18.6%; F = 29.9%; Figure 1). Compared to male intact dogs, male neutered dogs had an odds ratio of 2.17 (95% CI: 1.89–2.50; *p* < 0.001), indicating that male neutered dogs are 2.17 times more likely to receive a positive *BRAF* test result than male intact ones. Compared to their intact counterparts, neutered female dogs had an OR of 1.21 (95% CI: 1.05–1.38; *p* < 0.01).

**Figure 2:**
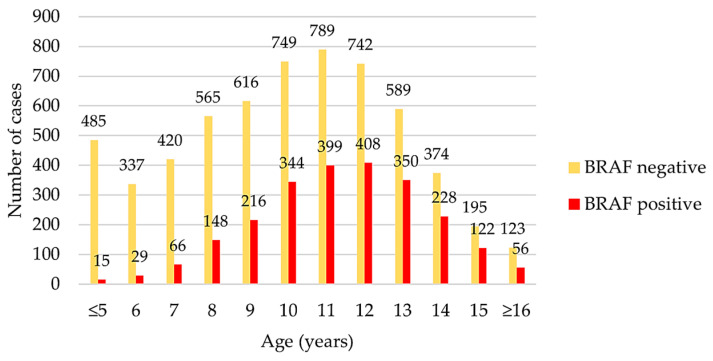
Diagram of the distribution by age and the results of the *BRAF* mutation analysis. The age of the dogs ranged between 1 and 23 years. The youngest dog with a *BRAF* mutation was less than 3 years old.

The mean age of all dogs tested in this cohort was 10.39 ± 2.93 years, with the youngest dog being 1 year old at diagnosis and the oldest dog being 23 years old. The age of the dogs diagnosed with a *BRAF* mutation was 11.4 ± 2.26 years (Figure 2). Our logistic regression analysis across all samples demonstrated that increasing age is associated with a higher risk of developing UC. When comparing the sample results of all dogs in this cohort, the study found an OR of 1.18 (95% CI: 1.16–1.20; *p* < 0.001), indicating that the probability of obtaining a positive *BRAF* result increases with the dog’s age. The *BRAF* mutation analysis was positive in 15 samples from dogs 5 years old or younger. The youngest dog, a female neutered Border Collie with a positive *BRAF* test result, was 2 years and eight months old. The other young dogs with the *BRAF* mutation were all 5 years old at the time of diagnosis. Among them were five mixed-breed dogs (one American Pitbull Terrier), two Scottish Terriers, and one Collie, Dobermann, Fox Terrier, Miniature Dachshund, Romaner Antikdogge, Shar Pei, and West Highland White Terrier (WHWT) each.

### 3.2. Data Set of the Most Common Breeds

**Figure 3:**
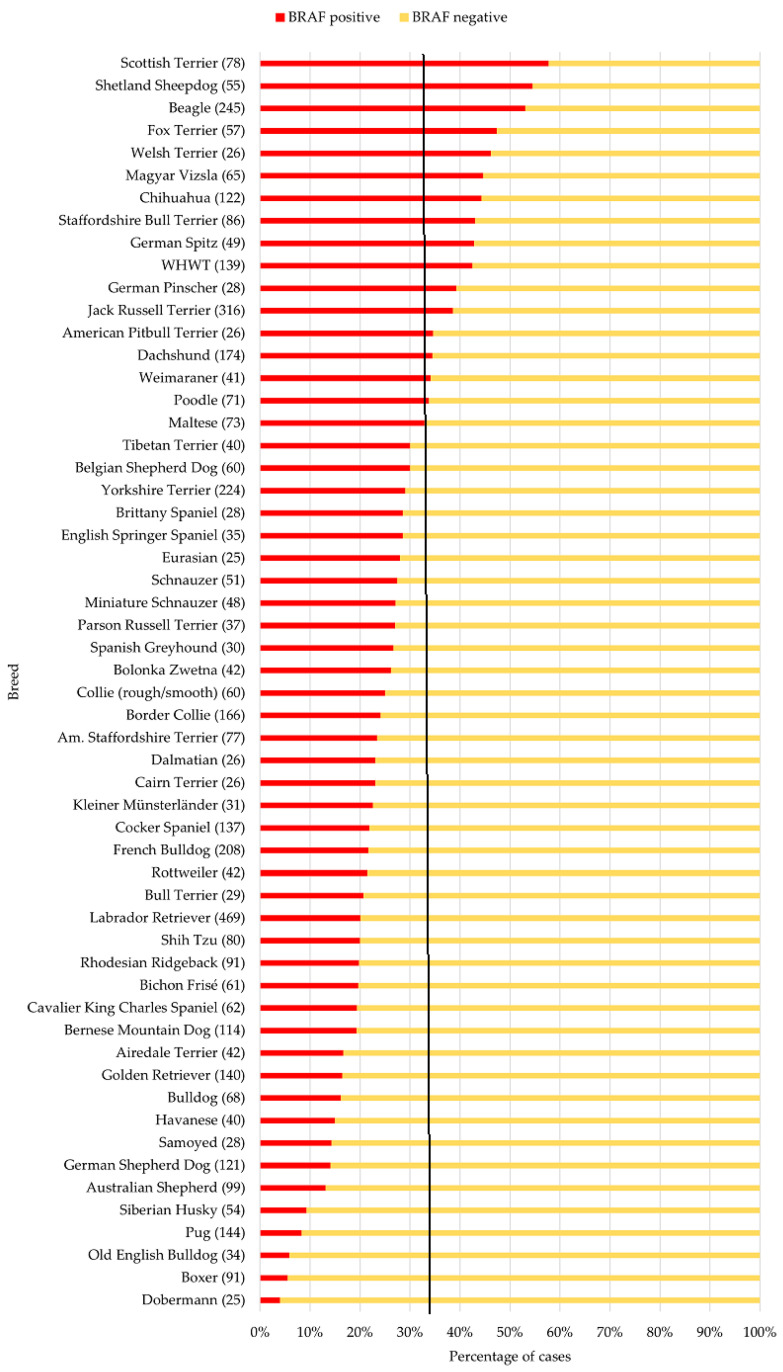
Diagram of the most common breeds (*n* ≥ 25) in the analysed sample material and their proportion of *BRAF*-positive and -negative test results compared with the proportions of the mixed breeds/not otherwise specified (black line, 33% positive cases), sorted in descending order according to the frequency of positive results. Total number of cases per breed in parentheses. Am. Staffordshire Terrier, American Staffordshire Terrier; WHWT, West Highland White Terrier.

To obtain an overview of the most common breeds in this study cohort, 56 breeds with *n* ≥ 25 cases were selected. In the mixed-breed dogs, the *BRAF* mutation was present in 774 samples (33%). The distribution of the *BRAF* results of the 56 individual breeds is summarised in Figure 3, sorted in descending order according to the frequency of positive results. The same distribution of these particular breeds, sorted in alphabetical order, is shown in Figure S1. The following will discuss only the *BRAF*-positive results in more detail, as the UC diagnosis is conclusive in these cases.

**Figure 4:**
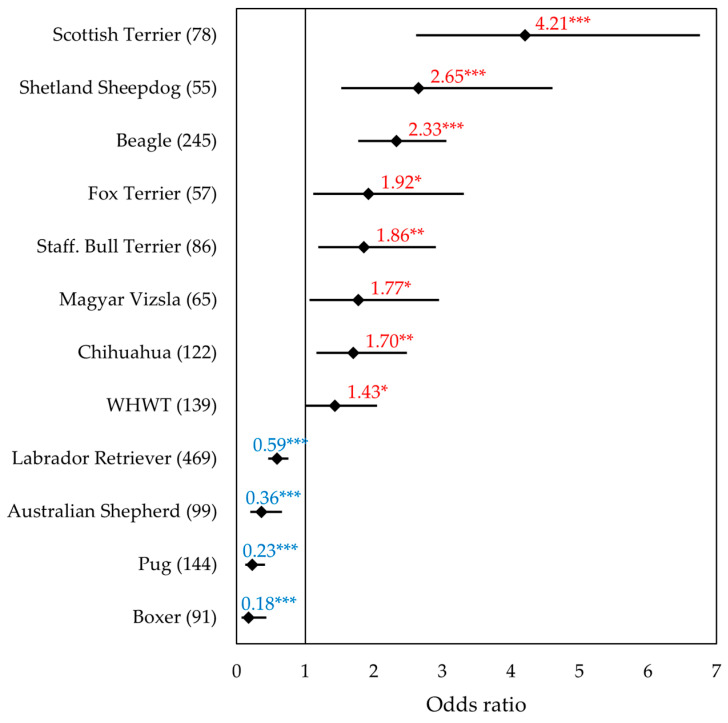
Forest plot of the odds ratios for ‘positive *BRAF* result’ of the 12 most common breeds based on the diagnostic data. The samples of these breeds are significantly more (red)/less (blue) *BRAF*-positive than the mixed breeds. Total number of cases per breed in parentheses. Significance: * *p* < 0.05, ** *p* < 0.01, *** *p* < 0.001; Staff. Bull Terrier, Staffordshire Bull Terrier; WHWT, West Highland White Terrier.

The ten breeds (*n* ≥ 25) with the highest proportion of *BRAF*-positive results included five terrier breeds (Scottish Terriers, Fox Terriers, Staffordshire Bull Terriers, Welsh Terriers, and WHWT) as well as Shetland Sheepdogs, Beagles, Magyar Vizslas, Chihuahuas, and German Spitz. In contrast, Boxers, Pugs, Australian Shepherds, and Labrador Retrievers showed a significantly reduced probability of a positive *BRAF* result compared to mixed-breed dogs. The results of multifactorial regression analysis are given in Figure 4. A detailed forest plot of the 56 individual breeds is shown in Figure S2.

The results of the samples from Scottish Terriers were noticeable. The OR of 4.21 showed that the *BRAF* mutation was detected more than four times as likely in samples from Scottish Terriers compared to mixed-breed dogs (95% CI: 2.62–6.76; *p* < 0.001). In our material, Shetland Sheepdogs had the second highest percentage of positive *BRAF* results (55%) after Scottish Terriers (58%). An odds ratio of 2.65 was found, indicating that samples from Shetland Sheepdogs were 2.65 times more likely to be positive for the *BRAF* mutation than the mixed-breeds (95% CI: 1.52–4.61; *p* < 0.001). Beagles were frequent in the cohort (*n* = 245); this breed also stood out in the percentage (53%) of positive *BRAF* test results. The OR of 2.33 showed that the *BRAF* mutation was more than twice as likely to be detected in samples from Beagles compared to mixed-breed dogs (95% CI: 1.77–3.06; *p* < 0.001). For Fox Terriers, the study showed an OR of 1.92 (95% CI: 1.12–3.31; *p* < 0.05), meaning that a positive *BRAF* test result was 1.92 times more likely in samples from Fox Terriers than mixed-breed dogs. A proportion of 43% of the samples from Staffordshire Bull Terriers had a positive *BRAF* result. This dog breed was 1.86 times more likely to be diagnosed with the *BRAF* mutation with this test than mixed-breed dogs (OR: 1.86; 95% CI: 1.19–2.91; *p* < 0.01).

In Magyar Vizslas, it is noticeable that over 60% of the samples from neutered male dogs had a positive *BRAF* result (63.2%). This dog breed is 1.77 times more likely to be diagnosed with the *BRAF* mutation with this test than mixed-breed dogs (OR: 1.77; 95% CI: 1.07–2.95; *p* < 0.05). The situation is similar with the samples from Chihuahuas in this cohort diagnosed with the *BRAF* mutation (OR: 1.70; 95% CI: 1.16–2.48; *p* < 0.01). For WHWTs, the study revealed that significantly more positive *BRAF* test results were expected compared to mixed breeds, as indicated by an odds ratio of 1.43 (95% CI: 1.00–2.05, *p* > 0.05). Welsh Terriers were relatively rare in the cohort (*n* = 28), with 46.2% *BRAF*-positive samples. There was no significant difference between the samples from Welsh Terriers and the mixed-breed dogs with regard to a positive *BRAF* result (OR: 1.71, 95% CI: 0.77–3.80, *p* = 0.19), which was probably due to the small number of cases. The logistic regression revealed no significant differences between a positive *BRAF* result in German Spitz and mixed-breed dogs (OR: 1.43, 95% CI: 0.79–2.57, *p* = 0.24).

**Figure 5:**
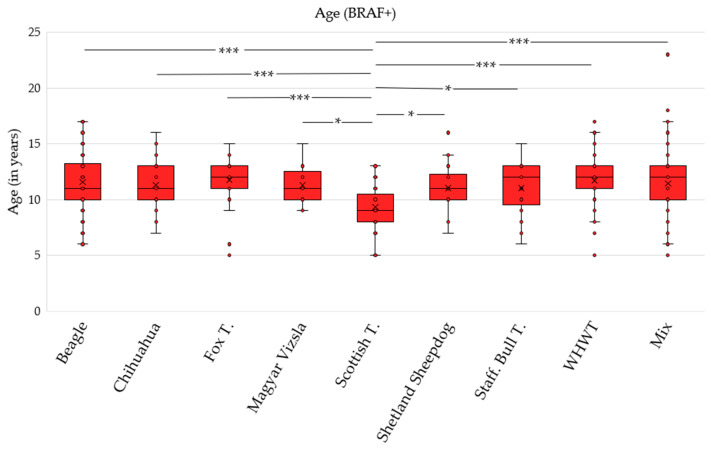
Boxplots of the age distribution of the breeds (*n* ≥ 55) with the highest proportion of *BRAF*-positive results compared to the mixed-breeds reference, sorted in alphabetical order. * *p* < 0.05; *** *p* < 0.001. Fox T., Fox Terrier; Scottish T., Scottish Terrier; Staff. Bull T., Staffordshire Bull Terrier; WHWT, West Highland White Terrier.

Concerning their age distribution, it is notable that even very young dogs from the age of 5 years on are affected in this cohort of the breeds with the highest proportion of *BRAF* mutations (Figure 5). When comparing the ages of these *BRAF*-positive dogs, significant differences were observed between breeds (*p* < 0.001). There is a significant difference between the age of Scottish Terriers with a positive *BRAF* result and that of the other breeds shown in the boxplot. On average, Scottish Terriers whose submitted samples tested positive for the *BRAF* mutation were around 2 years younger than dogs of the other breeds (1.69–2.41). Due to the small number of cases, the Welsh Terrier and German Spitz breeds are not included in these boxplots.

In contrast to the breeds described before, in samples from Labrador Retrievers, most of the results of the *BRAF* mutation analysis were negative (Figure 4). An OR of 0.59 was found for Labrador Retrievers, indicating that samples from this dog breed were significantly less likely to have a *BRAF* mutation than mixed breed dogs (95% CI: 0.46–0.76; *p* < 0.001). In samples from the Australian Shepherds (*n* = 99), it is noticeable that only one 11-year-old intact male dog in this study had a *BRAF* mutation. The OR was 0.36, indicating dogs of this breed are around three times less likely to test positive for *BRAF* than the mixed breed dogs (95% CI: 0.2–0.66; *p* < 0.001). Furthermore, only a few *BRAF*-positive cases were recorded in the group of Pugs. An OR of 0.23 indicated that they are around five times less likely to have a positive *BRAF* result than mixed-breed dogs (95% CI: 0.12–0.41; *p* < 0.001). Only five samples of 91 Boxers had a positive *BRAF* result. Boxers had an 82% lower probability of receiving a positive *BRAF* result, as indicated by an OR of 0.18 (95% CI: 0.07–0.44; *p* < 0.001).

### 3.3. Non-Terrier Breeds with the Highest and Lowest Proportion of BRAF-Positive Results

The non-terrier breeds with the highest and lowest *BRAF* mutation rates in this cohort are described in more detail below. The sex distribution of *BRAF*-positive cases differs between these breeds (Table 1). In general, the samples of the neutered dogs of both sexes had a significantly higher percentage of *BRAF* mutation than the samples of their intact counterparts. In Australian Shepherds, 97.3% (*n* = 36) of the intact male dogs had a negative *BRAF* result. Only one 11-year-old intact male showed the *BRAF* mutation.

Considering the age of these non-terrier dogs from the diagnostic data, a median age of 10 to 12 years was found at the time of diagnosis of the *BRAF* mutation (Table 2).

### 3.4. Data Set of the Terrier Breeds

**Figure 6:**
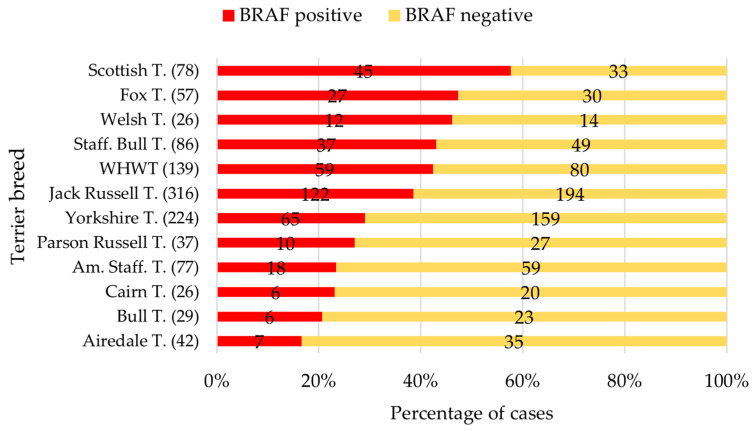
The most common terrier breeds (*n* > 25) and their proportion of positive *BRAF* results in red. Total number of cases per breed in parentheses. T., Terrier; Am. Staff. T., American Staffordshire Terrier; Staff. Bull T., Staffordshire Bull Terrier; WHWT, West Highland White Terrier.

Of all 1221 samples from the terrier cases, 36% (*n* = 440) were *BRAF*-positive. More than 40% of the samples from female terrier dogs were *BRAF*-positive (F: 42.9%; FN: 43.9%). The percentage of samples with *BRAF* mutations was lower in male terriers (M: 24.6%; MN: 37.5%). In total, our analysed data included samples from 47 terrier dogs aged between 1–5 years. Of these young terrier dogs, four samples tested positive for the *BRAF* mutation (2× Scottish Terrier, 1× Fox Terrier, 1× WHWT). The *BRAF* results for the terrier breeds *n* > 25 cases are shown in Figure 6. The sex distribution of the five terrier breeds with the highest proportion of *BRAF*-positive results is shown in Table 3.

**Figure 7:**
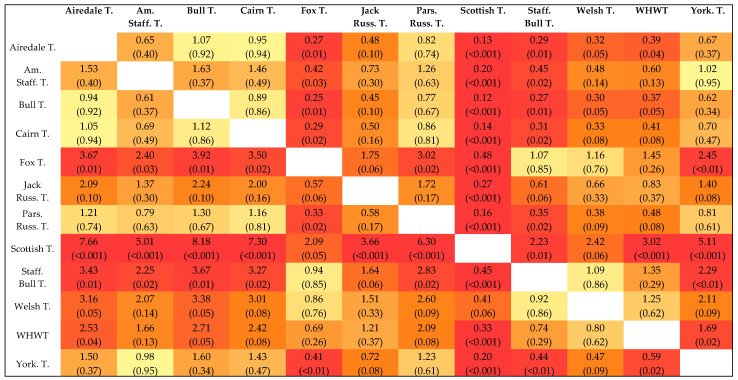
Odds ratio heatmap for terriers. For each of the 12 most common terrier breeds, the odds ratio for a positive *BRAF* result compared with the terrier breed in the respective column was calculated; the darker the colour (yellow to red), the smaller the *p*-value. Exact *p*-value in parentheses. T. = T., Terrier; Am. Staff. T., American Staffordshire Terrier; Jack Russ. T., Jack Russell Terrier; Par. Russ. T., Parson Russell Terrier; Staff. Bull T., Staffordshire Bull Terrier; WHWT, West Highland White Terrier; York. T., Yorkshire Terrier.

The Scottish Terrier had a unique position compared to mixed-breed dogs and to all other terrier breeds (Figure 6). For the terrier breeds (*n* > 25), except Welsh Terriers, the odds of receiving a positive *BRAF* result were significantly lower compared to Scottish Terriers (OR 0.12–0.48; all: *p* < 0.05; Figure 7).

Fox Terriers also had a significantly higher probability of being diagnosed with a *BRAF*-positive result than six other terrier breeds (*n* > 25).

Considering the age of these terriers from the diagnostic data, a median age of 12 years was found at the time of diagnosis of the *BRAF* mutation (Table 4). However, it is noticeable that the Scottish Terrier stood out with a median age of 9 years (range: 5–13 years).

## 4. Discussion

This study aimed to analyse the signalment of canine diagnostic samples for the *BRAF* mutation analysis submitted to LABOKLIN GmbH & Co. KG, Bad Kissingen, Germany. After selection according to the inclusion criteria, this sample material comprised 8365 samples, of which 2381 samples (28.5%) tested positive for the *BRAF* mutation. The data from across Europe and China were collected over six years (2018–2024), which provided a good overview of the diagnostic data of the *BRAF* test. This is the first study to analyse diagnostically submitted samples on this scale. An unavoidable source of bias in our sample set is that the material for the *BRAF* mutation analysis was submitted for (1) suspected UC, (2) to rule out UC in the presence of clinical symptoms, or (3) for screening purposes. Thus, it gives no data about sensitivity of the test because we have no information about the final diagnosis. The limitations of the test results must be pointed out: Only a positive *BRAF* test result is diagnostic, and reliable conclusions can be drawn from these data. As the *BRAF* mutation analysis has a specificity of 100% [28,30,33], it can be assumed that all dogs with a *BRAF* mutation were also diagnosed with UC.

It must be noticed that there is a natural limitation of this V595E mutation as a molecular marker for UC in dogs, as approximately 20% of tumours do not have the mutation [28,29,30]. Furthermore, many factors may influence the decision to conduct *BRAF* mutation analysis. Due to the lack of clinical data, no conclusions about sensitivity can be extracted from the *BRAF*-negative cases of this study.

However, the results of the present study are based on material submitted to LABOKLIN by partners, especially throughout Europe. The few samples from China showed a similar breed and *BRAF* result distribution compared to those of European dogs. Therefore, the data situation in the USA, where the Breen-Lab (Dept. of Molecular Biomedical Sciences, College of Veterinary Medicine, NC State University) developed the ddPCR-based *BRAF* V595E assay, may be different [30]. It would be interesting to compare the data from Europe and the USA.

On the one hand, some clinical studies have compared the frequency of UC in different dog breeds. There is a breed-specific risk of UC in dogs, especially for Scottish Terriers, Shetland Sheepdogs, Beagles, Fox Terriers, and WHWTs [4,7]. The cause of this risk in predisposed breeds is still unknown; however, it most likely reflects a genetic tendency for bladder cancer, such as variations in the biochemical processes that activate and eliminate carcinogens [4,7].

On the other hand, some studies have analysed UC in dogs for the *BRAF* mutation and have shown a breed-specific predisposition for terrier breeds. Interestingly, this predisposition for the *BRAF* mutation in terriers also corresponds to the frequent diagnosis of UC compared to other breeds. Aupperle et al. analysed samples from dogs with confirmed diagnostic UC for the *BRAF* mutation [32]. The sample material (biopsies, urine samples, cytological smears) from 116 dogs with confirmed UC was analysed. The cohort comprised 28 terrier dogs, mostly Jack Russell Terriers (*n* = 8). The canine *BRAF* V595E mutation was present in 24 out of 28 (86%) dogs of the terrier breeds. The study by Pantke (2019) also found in a smaller patient population that terriers have a significantly higher prevalence of the *BRAF* mutation compared to the other breeds [31]. In the American literature, Scottish Terriers were the most common dog breed in the cohort for *BRAF*-positive UC [4,7]. In contrast, in the study by Decker et al., no predisposition to the *BRAF* mutation was detected in UC of terrier breeds (*n* = 18) compared to the other breeds (*n* = 38) [28].

In our study, the *BRAF*-positive cases reflect the findings already described in the published literature. Given that a *BRAF* mutation confirms the presence of UC, we characterized the population of these dogs in more detail.

In this study’s cohort, the dog’s age (11.4 ± 2.26 years) was similar to that of previous studies [3,7]. Interestingly, our study detected the *BRAF* mutation in 15 samples from dogs aged 5 years and younger. Therefore, a screening test can be recommended from the age of 5 years, especially in predisposed breeds.

This study reveals that the probability of being diagnosed with a positive *BRAF* result and thus being diagnosed with UC was almost two times higher (OR: 1.88; 95% CI: 1.62–2.17; *p* < 0.001) in intact female dogs than in intact males. Although we do not know anything about the *BRAF*-negative cases in our cohort, this outcome is similar to the study by Knapp et. al, who determined an odds ratio of 1.96 with a 95% CI of 1.67–2.30 [7]. In three breeds (Shetland Sheepdog, Australian Shepherd, and WHWT), samples from intact females tested *BRAF*-positive significantly more often than those from intact males. This finding suggests a breed-specific predisposition or clustering of positive cases among intact female dogs. It is challenging to discuss the influence of castration, the reason for castration, or the time between castration and the sampling time for *BRAF* mutation analysis. Therefore, the results of the comparison of intact and neutered males and females should be interpreted with caution. From our data, it may be concluded that neutered dogs of both sexes have an increased risk of the *BRAF* mutation compared to intact dogs of the same sex. Our study revealed that male neutered dogs have a 2.17-fold and female neutered dogs a 1.21-fold higher probability of being diagnosed with a *BRAF* mutation than their counterparts. The OR of 4.08 for neutered males and 4.52 for neutered females was reported, indicating an increased risk of bladder cancer associated with neuter status [7]. In contrast, Aupperle et al. could not determine a sex-specific predisposition for detecting the *BRAF* mutation [32]. However, four of the breeds we examined in this study (Shetland Sheepdog, Labrador Retriever, Australian Shepherd, and WHWT) showed a significant difference in *BRAF* test results between samples from intact and neutered males. Samples from neutered males were significantly more often *BRAF*-positive than those from intact males. Among German Spitz, neutered females were significantly more likely to be diagnosed with UC compared to their intact counterparts. These results of this study suggest a breed-specific predisposition for neutered dogs to test *BRAF*-positive.

The present cohort confirmed the breed predisposition for the *BRAF* mutation in terrier breeds, which has already been described previously [32]. Out of 1221 samples from terriers, 36% (*n* = 440) were *BRAF*-positive. It should be noted that the sample material submitted for our study includes not only patients with a high suspicion of having UC—such as those with a mass in the urinary bladder on ultrasound or with clinical symptoms of urinary tract disease—but also cases submitted for screening purposes or to rule out UC for other reasons. Thus, the percentage of positive cases is lower in this study than in studies that only depend on certain specific UC material [7,31,32]. However, it is evident that samples from the four terrier breeds, Scottish Terrier, Fox Terrier, Staffordshire Bull Terrier, and West Highland White Terrier (WHWT), are significantly more likely to be diagnosed with a *BRAF* mutation compared with mixed-breed dogs (OR 1.43–4.21). These terrier breeds were also pointed out in the study by Mutsaers et al. (2003) [4].

Our study’s high number of *BRAF*-positive cases is consistent with the existing breed predisposition for UC in Scottish Terriers. Knapp et al. found a 19-fold increased risk of UC in Scottish Terriers compared to mixed breeds [7]. The Scottish Terrier also stood out significantly in our internal comparison of terrier breeds: with a 2- to 8-fold increased probability of receiving a positive *BRAF* result, the Scottish Terrier was predisposed considerably compared to the other terrier breeds. Scottish Terriers diagnosed with a *BRAF* mutation were significantly younger than other breeds with a high percentage of positive *BRAF* results. It is important to note that the domestic dog is one of the most diverse mammalian species in terms of breed-related differences in morphology and morbidity [34,35]. Across dog breeds, longevity is negatively correlated with body size—larger breeds tend to have shorter life expectancies than smaller or miniature breeds [1,36]. Additionally, mixed-breed dogs generally live longer than purebred dogs, regardless of body weight [37].

In the present study, it was noticeable that very few *BRAF*-positive cases (12/144) among the sample material were submitted from Pugs, and the logistic regression also showed a significantly lower probability of obtaining a positive *BRAF* result for this breed. Due to the small size of the dog breed and the limitations of invasive sampling (at-risk patients for anaesthesia), it may be hypothesized that the *BRAF* mutation analysis of urine samples was used early in the diagnostic workup of such patients.

**Figure 8:**
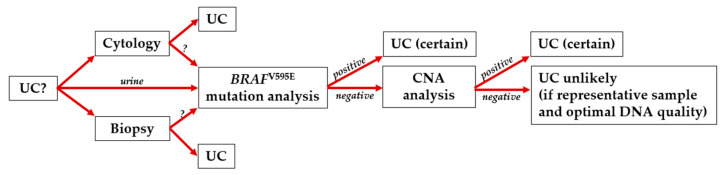
Decision flow diagram for the procedure in case of suspected urothelial carcinoma (UC). Molecular technique enables reliable UC diagnosis of representative samples with high DNA quality in case of a positive test result from urine DNA.

The sensitivity of the ddPCR assay for the V595E mutation is limited to around 80% [30]. For *BRAF*-negative cases, a further diagnostic step can be used with the already isolated DNA to increase sensitivity to diagnose UC by the copy number alterations (CNA) on chromosomes 13, 19, and 36 [16,38,39]. This additional test is offered as ‘BRAF Complete’ by LABOKLIN GmbH & Co. KG in Europe and ‘CADET BRAF-PLUS’ by Antech in the USA. If the CNA analysis is positive, UC can be diagnosed with certainty (Figure 8). However, it must be noted that some DNA samples are suitable for *BRAF* mutation analysis, but not for CNA analysis. Especially other cells than urothelium (inflammation, bacteria, sperm) can lead to false negative results. Furthermore, a better DNA quality is necessary because more extended sequences have to be analysed compared to the *BRAF* gene point-mutation.

In summary, the analysis of the higher number of diagnostic samples showed that molecular genetic test results clearly added value in the workflow for both the clinician and the animal. Molecular genetic analysis for the canine *BRAF* V595E mutation is another tool for diagnosing UC. Considering the predisposed dog breeds, UC can be diagnosed by *BRAF* mutation analysis in the case of nonspecific lower urinary tract symptoms. Minimally invasive sample collection, low costs for patient owners, and no risk of anaesthesia in older or pre-diseased patients are clear advantages. In addition, this technique may be helpful in cases with questionable cytological or histological findings, such as those showing malignant criteria that are not clearly identified as neoplastic, or cases with an insufficient number of cells to establish a definitive diagnosis. A case report described the detection of the *BRAF* V595E mutation via digital PCR in a urine sample from a dog with follicular cystitis and a flat urothelial lesion exhibiting atypia [40]. This finding suggests that the mutation may represent an early driver event in the progression from dysplasia to carcinoma.

The role and potential of *BRAF* inhibitors as a therapeutic option for human cancer are of high importance [41,42]. Furthermore, it should be noted that *BRAF*-targeted therapy in veterinary medicine for canine UC has been increasingly researched in recent studies [28,43,44]. The human V600E variant was used as a model, and since vemurafenib suppresses MAPK/ERK kinase (MEK) activation, it was tested whether this drug can inhibit the proliferation of canine UC cells. Phosphorylated MEK levels were elevated in three *BRAF* V595E mutant cell lines (K9TCC-An, AxA, and Nk) compared to one wild-type cell line (K9TCC-Sh), and could be significantly reduced by vemurafenib [28]. Two of the lines (K9TCC-An and AxA) showed a marked decrease in proliferation in response to treatment [28]. Therefore, the *BRAF* inhibitors may represent a therapeutic option for *BRAF*-positive canine UC. Another study has shown that UC cells with *BRAF* mutation react more sensitively to sorafenib than to vemurafenib [44]. Tolerability has even been reported with oral application of sorafenib [45].

*BRAF* mutation analysis, as a third diagnostic option alongside cytological and histopathological examination of unconfirmed masses in the canine urothelial tract, is important for accurately determining a diagnosis of UC even at an early stage (Figure 8). From a clinical perspective, identifying a *BRAF*-positive UC can help to guide treatment decisions and may contribute to patient outcomes and prolonged survival time. Taken together, we can conclude that the additional diagnostic tool of a *BRAF* mutation analysis, considering predisposing factors such as the dog breed in particular, is a quick and straightforward way to a possible UC diagnosis and has additional therapeutic impact (*BRAF* inhibitors).

## 5. Conclusions

The results of this retrospective study on diagnostic samples for *BRAF* mutation analysis show that the breed predispositions for UC are also reflected in the positive *BRAF* results. Therefore, the analysed statistical data regarding the signalment can be used to decide which patients (breed, sex, age) will most likely profit from this investigation. Importantly, *BRAF* mutation screening for the predisposed dog breeds is already advisable at the age of 5 years.

## Figures and Tables

**Table 1:** Non-terrier breeds with the highest and lowest (grey shadow) proportion of *BRAF*-positive results and their sex distribution. The breeds are sorted in descending order according to the percentage of *BRAF*-positive results.

Breed (*n; BRAF*+ in %)	M	MN	F	FN	Significance *p*
	*n* (*BRAF*+/Total) *BRAF*+ in %				
Shetland Sheepdog (55; 54.5%)	4/17	8/10	8/12	10/16	a < 0.01 *, b = 0.03 *
	23.5 ^a,b^	80.0 ^a^	66.7 ^b^	62.5	
Beagle (245; 53.1%)	21/55	29/57	34/61	46/72	n.s.
	38.2	50.9	55.7	63.9	
Magyar Vizsla (65; 44.6%)	6/17	12/19	5/10	6/19	n.s.
	35.3	63.2	50.0	31.6	
Chihuahua (122; 44.3%)	9/35	14/30	11/23	20/34	n.s.
	25.7	46.7	47.8	58.8	
German Spitz (49; 42.9%)	2/9	7/13	2/12	10/15	a < 0.01 *
	22.2	53.8	16.7 ^a^	66.7 ^a^	
Labrador Retriever (469; 20.0%)	20/151	34/130	16/85	24/103	a < 0.01 *
	13.2 ^a^	26.1 ^a^	18.8	23.3	
Australian Shepherd (99; 13.1%)	1/37	5/17	4/18	3/27	a < 0.01 *, b = 0.04 *
	2.7 ^a,b^	29.4 ^a^	22.2 ^b^	11.1	
Pug (144; 8.3%)	4/32	2/13	2/41	4/58	n.s.
	12.5	15.4	4.9	6.9	
Boxer (91; 5.5%)	2/41	0/19	2/17	1/14	n.s.
	4.9	0	11.8	7.1	

Abbreviations: M, male neutered; MN, male neutered; F, female; FN, female neutered; a and b indicate statistical comparisons between the two marked sex groups within the breed; significance: * *p* < 0.05 of chi-squared or Fisher’s exact test, respectively; n.s., not significant.

**Table 2:** Non-terrier breeds with the highest and lowest (grey shadow) proportion of *BRAF*-positive results and their age distribution (median age of all samples, of *BRAF*-positive and *BRAF*-negative samples), sorted in descending order according to the submitted case numbers.

Breed (*n*)	Age (Total)	Age (*BRAF*+)	Age (*BRAF*−)
	Range (Median) in Years		
Shetland Sheepdog (55)	6–16 (11)	7–16 (11)	6–16 (11)
Beagle (245)	2–17 (11)	6–17 (11)	2–17 (11)
Magyar Vizsla (65)	4–15 (11)	9–15 (11)	4–15 (11)
Chihuahua (122)	1–16 (11)	7–16 (11)	1–16 (11)
German Spitz (49)	5–18 (11)	9–17 (12)	5–18 (11)
Labrador Retriever (469)	1–18 (10)	6–16 (11)	1–18 (10)
Australian Shepherd (99)	1–15 (10)	7–14 (11)	1–15 (10)
Pug (144)	3–16 (9)	8–14 (10)	3–16 (9)
Boxer (91)	1–14 (9)	8–10 (10)	1–14 (9)

**Table 3:** Terrier breeds with the highest proportion of *BRAF* mutations and their sex distribution. The breeds are sorted in descending order according to the percentage of *BRAF*-positive results.

Breed (*n; BRAF*+ in %)	M	MN	F	FN	Significance *p*
	*n* (*BRAF*+/Total) *BRAF*+ in %				
Scottish Terrier (78; 57.7%)	14/32	3/6	15/22	13/18	n.s.
	43.8	50.0	68.2	72.2	
Fox Terrier (57; 47.4%)	5/21	10/20	4/6	8/10	n.s.
	23.8	50.0	66.7	80.0	
Welsh Terrier (26; 46.2%)	2/6	2/8	2/4	6/8	n.s.
	33.3	25.0	50.0	75.0	
Staff. Bull Terrier (86; 43.0%)	9/30	9/18	5/15	14/23	n.s.
	30.0	50.0	33.3	60.9	
WHWT (139; 42.4%)	7/35	9/18	22/42	21/44	a = 0.02 *, b < 0.01 *
	20.0 ^a,b^	50.0 ^a^	52.4 ^b^	47.7	

Abbreviations: M, male neutered; MN, male neutered; F, female; FN, female neutered; a and b indicate statistical comparisons between the two marked sex groups within the breed; significance: * *p* < 0.05 of chi-squared or Fisher’s exact test, respectively; n.s., not significant. Staff. Bull Terrier, Staffordshire Bull Terrier; WHWT, West Highland White Terrier.

**Table 4:** Terrier breeds with the highest proportion of *BRAF*-positive results and their age distribution (median age of all samples, of *BRAF*-positive samples, and of *BRAF*-negative samples), sorted in descending order according to the percentage of *BRAF*-positive results.

Breed (*n*)	Age (Total)	Age (*BRAF*+)	Age (*BRAF*−)
	Range (Median) in Years		
Scottish Terrier (78)	4–13 (10)	5–13 (9)	4–13 (10)
Fox Terrier (57)	3–16 (12)	5–15 (12)	3–16 (11)
Welsh Terrier (26)	1–16 (12)	9–16 (12)	1–14 (11)
Staff. Bull Terrier (86)	4–15 (11)	6–15 (12)	4–14 (10)
WHWT (139)	1–17 (12)	5–17 (12)	1–17 (12)

Abbreviations: Staff. Bull Terrier, Staffordshire Bull Terrier; WHWT, West Highland White Terrier.

## Data Availability

The original contributions presented in the study are included in the article/Supplementary Materials; further inquiries can be directed to the corresponding author/s.

## Supplementary Materials

The following supporting information can be downloaded at: https://www.mdpi.com/article/10.3390/vetsci12080729/s1. Figure S1: *BRAF* result distribution of the most common breeds (n ≥ 25), sorted in alphabetical order; Figure S2: Forest plot of the odds ratios for ‘positive *BRAF* result’ of the 56 individual breeds; Table S1: Information for all included dogs tested for the *BRAF* mutation V595E with breed, age at the time of sampling, sex, sample material, and *BRAF* test result.

## Abbreviations

The following abbreviations are used in this manuscript:

CNA	Copy number alterations
ddPCR	Droplet digital PCR
FCI	Fédération Cynologique Internationale
FFPE	Formalin-fixed, paraffin-embedded
CI	Confidence interval
MAPK/ERK	Mitogen-activated protein kinase/extracellular regulated kinase
MEK	MAPK/ERK kinase
OR	Odds ratio
*p*	*p*-value
UC	Urothelial carcinoma
WHWT	West Highland White Terrier
