# Supplementary material for: BRAF Mutation Analysis: A Retrospective Evaluation of 8365 Diagnostic Samples with a Special View on Canine Breeds (2018–2024)
Source: Vet Sci. 2025 Aug 2;12(8):729. doi: 10.3390/vetsci12080729 (PMC12390318; doi:10.3390/vetsci12080729)
Supplement: Supplementary file 1 [file vetsci-12-00729-s001.zip › Figure S1.pdf]

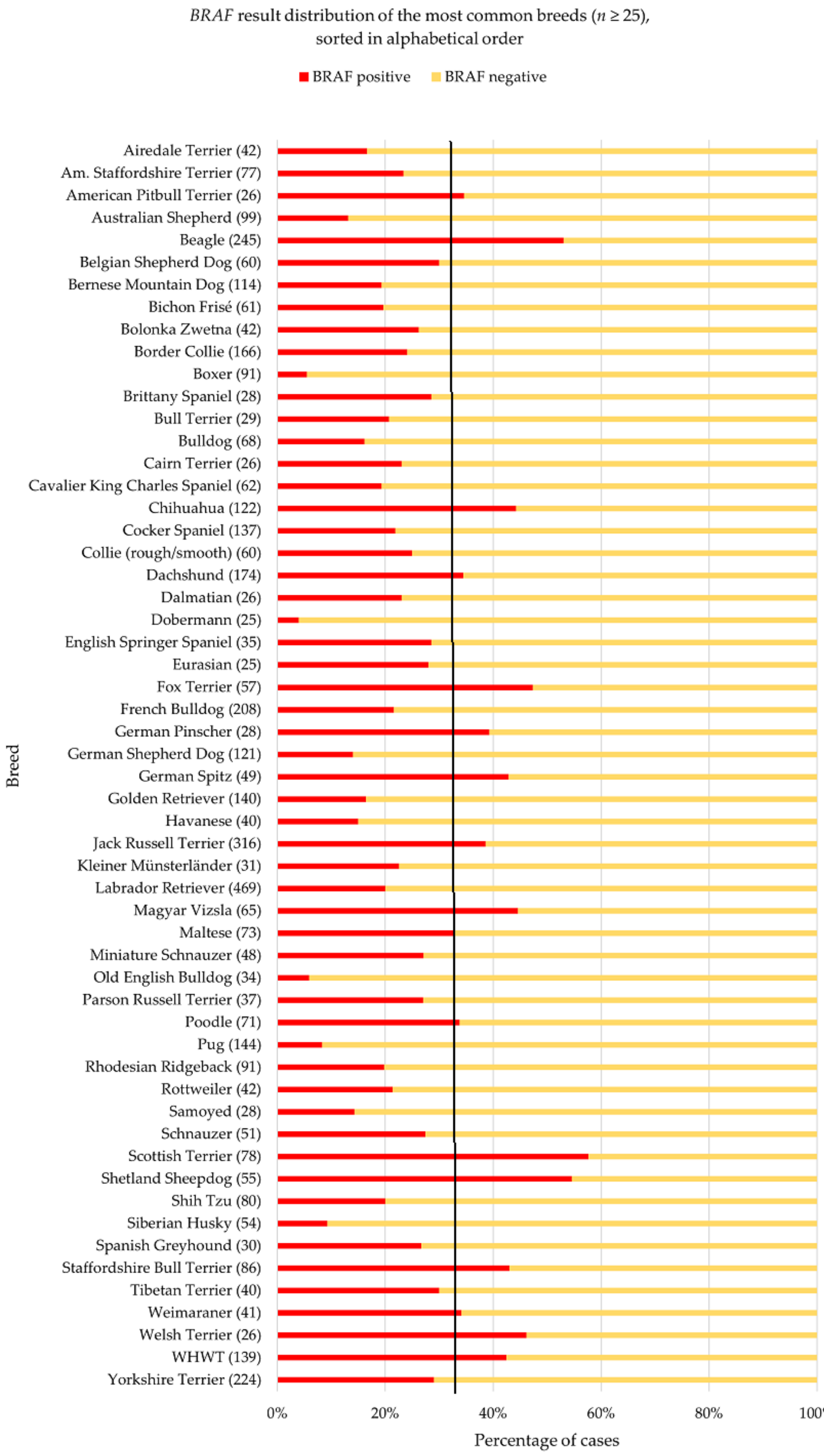

Figure S1. Diagram of the most common breeds in the analysed sample material and their proportion of *BRAF*-positive and -negative test results compared with the proportions of the mixed breeds/not otherwise specified (black line, 33% positive cases); sorted in alphabetical order. Total number of cases per breed in parentheses. Am. Staffordshire Terrier, American Staffordshire Terrier; WHWT, West Highland White Terrier.
