# Supplementary material for: BRAF Mutation Analysis: A Retrospective Evaluation of 8365 Diagnostic Samples with a Special View on Canine Breeds (2018–2024)
Source: Vet Sci. 2025 Aug 2;12(8):729. doi: 10.3390/vetsci12080729 (PMC12390318; doi:10.3390/vetsci12080729)
Supplement: Supplementary file 1 [file vetsci-12-00729-s001.zip › Figure S2.pdf]

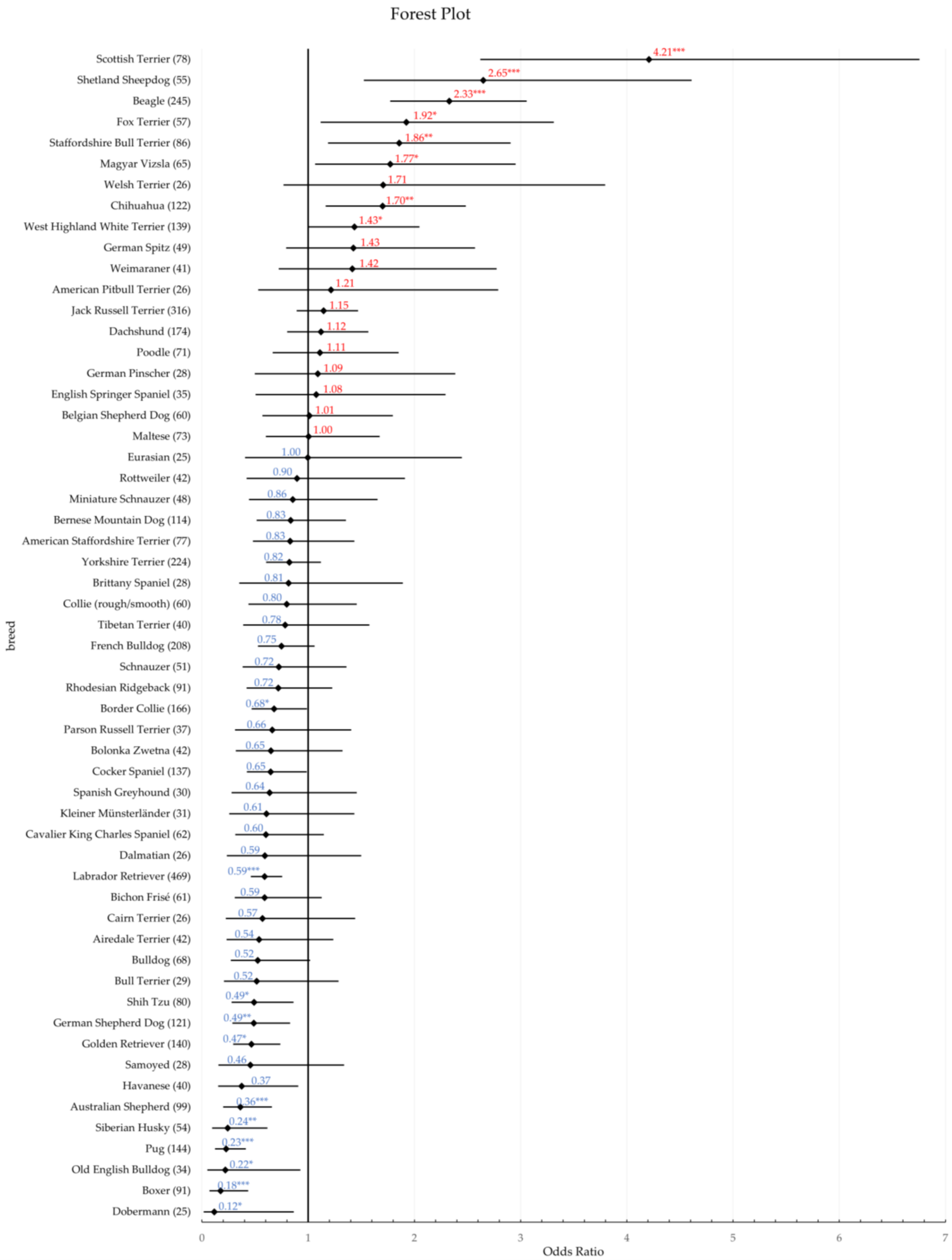

Figure S2: Forest plot of the odds ratios for `positive BRAF result' of the 56 individual breeds. The samples of these breeds are significantly more (red)/ less (blue) *BRAF*-positive compared to the mixed breeds. Total number of cases per breed in parentheses; significance: *p < 0.05, **p < 0.01, ***p < 0.001.
